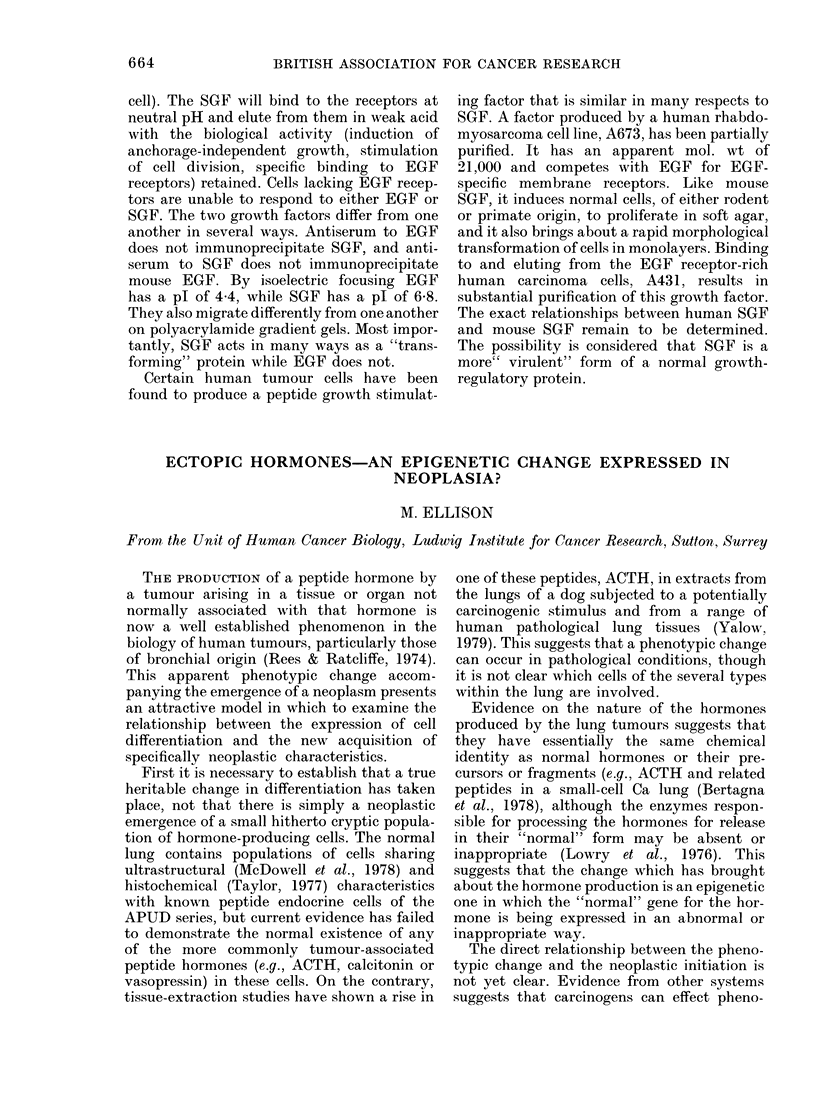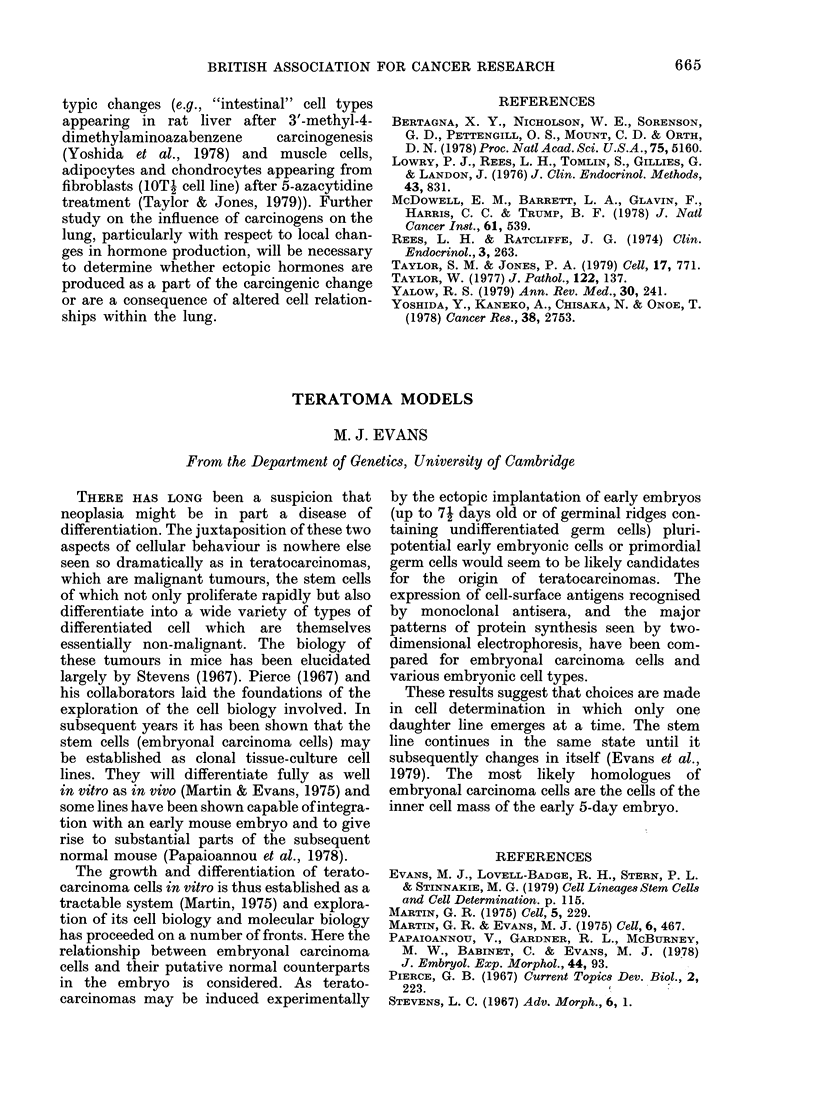# Ectopic Hormones—An Epigenetic Change Expressed in Neoplasia?

**Published:** 1980-04

**Authors:** M. Ellison


### ECTOPIC HORMONES—AN EPIGENETIC CHANGE EXPRESSED IN NEOPLASIA?

M. ELLISON

*From the Unit of Human Cancer Biology, Ludwig Institute for Cancer Research, Sutton, Surrey*

The production of a peptide hormone by a tumour arising in a tissue or organ not normally associated with that hormone is now a well established phenomenon in the biology of human tumours, particularly those of bronchial origin (Rees & Ratcliffe, 1974). This apparent phenotypic change accompanying the emergence of a neoplasm presents an attractive model in which to examine the relationship between the expression of cell differentiation and the new acquisition of specifically neoplastic characteristics.

First it is necessary to establish that a true heritable change in differentiation has taken place, not that there is simply a neoplastic emergence of a small hitherto cryptic population of hormone-producing cells. The normal lung contains populations of cells sharing ultrastructural (McDowell *et al.*, 1978) and histochemical (Taylor, 1977) characteristics with known peptide endocrine cells of the APUD series, but current evidence has failed to demonstrate the normal existence of any of the more commonly tumour-associated peptide hormones (*e.g.*, ACTH, calcitonin or vasopressin) in these cells. On the contrary, tissue-extraction studies have shown a rise in one of these peptides, ACTH, in extracts from the lungs of a dog subjected to a potentially carcinogenic stimulus and from a range of human pathological lung tissues (Yalow, 1979). This suggests that a phenotypic change can occur in pathological conditions, though it is not clear which cells of the several types within the lung are involved.

Evidence on the nature of the hormones produced by the lung tumours suggests that they have essentially the same chemical identity as normal hormones or their precursors or fragments (*e.g.*, ACTH and related peptides in a small-cell Ca lung (Bertagna *et al.*, 1978), although the enzymes responsible for processing the hormones for release in their "normal" form may be absent or inappropriate (Lowry *et al.*, 1976). This suggests that the change which has brought about the hormone production is an epigenetic one in which the "normal" gene for the hormone is being expressed in an abnormal or inappropriate way.

The direct relationship between the phenotypic change and the neoplastic initiation is not yet clear. Evidence from other systems suggests that carcinogens can effect phenotypic changes (*e.g.*, "intestinal" cell types appearing in rat liver after 3′-methyl-4-dimethylaminoazabenzene carcinogenesis (Yoshida *et al.*, 1978) and muscle cells, adipocytes and chondrocytes appearing from fibroblasts (10T½ cell line) after 5-azacytidine treatment (Taylor & Jones, 1979)). Further study on the influence of carcinogens on the lung, particularly with respect to local changes in hormone production, will be necessary to determine whether ectopic hormones are produced as a part of the carcingenic change or are a consequence of altered cell relationships within the lung.

REFERENCES

BERTAGNA, X. Y., NICHOLSON, W. E., SORENSON, G. D., PETTENGILL, O. S., MOUNT, C. D. & ORTH, D. N. (1978) *Proc. Natl Acad. Sci. U.S.A.*, **75**, 5160.

LOWRY, P. J., REES, L. H., TOMLIN, S., GILLIES, G. & LANDON, J. (1976) *J. Clin. Endocrinol. Methods*, **43**, 831.

McDOWELL, E. M., BARRETT, L. A., GLAVIN, F., HARRIS, C. C. & TRUMP, B. F. (1978) *J. Natl Cancer Inst.*, **61**, 539.

REES, L. H. & RATCLIFFE, J. G. (1974) *Clin. Endocrinol.*, **3**, 263.

TAYLOR, S. M. & JONES, P. A. (1979) *Cell*, **17**, 771.

TAYLOR, W. (1977) *J. Pathol.*, **122**, 137.

YALOW, R. S. (1979) *Ann. Rev. Med.*, **30**, 241.

YOSHIDA, Y., KANEKO, A., CHISAKA, N. & ONOE, T. (1978) *Cancer Res.*, **38**, 2753.